# Phenolic compounds as Nrf2 inhibitors: potential applications in cancer therapy

**DOI:** 10.1186/s12964-023-01109-0

**Published:** 2023-05-01

**Authors:** Javad Sharifi-Rad, Veronique Seidel, Michalak Izabela, Margalida Monserrat-Mequida, Antoni Sureda, Valeska Ormazabal, Felipe A. Zuniga, Shivaprasad Shetty Mangalpady, Raffaele Pezzani, Alibek Ydyrys, Gulmira Tussupbekova, Miquel Martorell, Daniela Calina, William C. Cho

**Affiliations:** 1 Facultad de Medicina, Universidad del Azuay, Cuenca, Ecuador; 2 Natural Products Research Laboratory, Strathclyde Institute of Pharmacy and Biomedical Sciences, University of Strathclyde, Glasgow, UK; 3 Department of Advanced Material Technologies, Faculty of Chemistry, Wroclaw University of Science and Technology, Smoluchowskiego 25, 50-372 Wroclaw, Poland; 4 Research Group in Community Nutrition and Oxidative Stress, University of the Balearic Islands—IUNICS, 07122 Palma, Spain; 5 Health Research Institute of Balearic Islands (IdISBa), 07120 Palma, Spain; 6 CIBER Fisiopatología de La Obesidad Y Nutrición (CIBEROBN), Instituto de Salud Carlos III (ISCIII), 28029 Madrid, Spain; 7 Department of Pharmacology, Faculty of Biological Sciences, University of Concepción, Concepción, Chile; 8 Department of Clinical Biochemistry and Immunology, Faculty of Pharmacy, University of Concepción, Concepción, Chile; 9 Department of Chemistry, NMAM Institute of Technology, Nitte, Karnataka 574110 India; 10 Phytotherapy Lab, Endocrinology Unit, Department of Medicine (DIMED), University of Padova, Via Ospedale 105, 35128 Padova, Italy; 11 AIROB, Associazione Italiana Per La Ricerca Oncologica Di Base, Padova, Italy; 12 Biomedical Research Centre, Al-Farabi Kazakh National University, Al-Farabi Ave. 71, 050040 Almaty, Kazakhstan; 13 The Elliott School of International Affairs, 1957 E St NW, George Washington UniversityWashington DC, 20052 USA; 14 Department of Biophysics, Biomedicine and Neuroscience, Al-Farabi Kazakh National University, Al-Farabi Ave. 71, 050040 Almaty, Kazakhstan; 15 Department of Nutrition and Dietetics, Faculty of Pharmacy, and Centre for Healthy Living, University of Concepción, Concepción, Chile; 16 Universidad de Concepción, Unidad de Desarrollo Tecnológico, UDT, 4070386 Concepción, Chile; 17 Department of Clinical Pharmacy, University of Medicine and Pharmacy of Craiova, 200349 Craiova, Romania; 18 Department of Clinical Oncology, Queen Elizabeth Hospital, Kowloon, Hong Kong

**Keywords:** Phenolic compounds, Cancer, Nrf2, Oxidative stress, Cytotoxicity, Apoptosis

## Abstract

Cancer is a leading cause of death worldwide and involves an oxidative stress mechanism. The transcription factor Nrf2 has a crucial role in cytoprotective response against oxidative stress, including cancer growth and progression and therapy resistance. For this reason, inhibitors of Nrf2 are new targets to be studied. Traditional plant-based remedies rich in phytochemicals have been used against human cancers and phenolic compounds are known for their chemopreventive properties. This comprehensive review offers an updated review of the role of phenolic compounds as anticancer agents due to their action on Nrf2 inhibition. In addition, the role of naturally-occurring bioactive anticancer agents are covered in the clinical applications of polyphenols as Nrf2 inhibitors.

Video Abstract

**Supplementary Information:**

The online version contains supplementary material available at 10.1186/s12964-023-01109-0.

## Introduction

Each year millions of people suffer from different types of cancer and almost half of them die due to the progression of this to an uncontrollable condition [1]. Cancer is the second leading cause of mortality globally, with an estimated 19 million new cases and 10 million deaths yearly in 2020 [2].

Tumors are composed of heterogeneous cells with the capacity to adapt dynamically their microenvironment by genetic/epigenetic changes and metabolic reprogramming [3, 4]. The tumour cells are formed in the body due to metastasis, uncontrolled proliferation, lower immunity in the body, and resistance to apoptosis [5]. Apart from this, a higher level of oxidative stress exerted by reactive oxygen species (ROS) and/or reactive nitrogen species (RNS) is one of the major features of cancerous cells [6–8]. In humans, complex protective machinery defends against the attacks of ROS and RNS, which are regularly produced in the human body as a result of cellular metabolism and environmental exposure [9, 10]. The metabolic rewiring promotes cancer cell proliferation and tumor growth. Moreover, an increased ROS production is counterbalanced by an increased endogenous antioxidant capacity as part of the adaptive response by cancer cells to face adverse conditions [11]. Cancer cells during malignant progression can often develop resistance to treatment [12, 13]. The increased antioxidant capacity of cancer cells is a crucial determinant of resistance to therapy [14]. In this sense, inhibiting the metabolic and antioxidant circuits that support the redox balance in cancer cells may be a promising anticancer therapy. Especially considering that conventional anticancer therapies generate cytotoxicity dependent on the efficient accumulation of ROS [14, 15]. The nuclear factor E2-related factor 2 (Nrf2)/Kelch-like ECH-associated protein 1 (Keap1) signalling pathway is one of the most important for cell defence against xenobiotic and oxidative stress [16, 17].

In normal cells, Nrf2 plays a crucial role in the cellular defense mechanism that protects cells and promotes cell survival under stress conditions [18]. Additionally, it is a tumor suppressor that can remove ROS and carcinogenic agents [19]. However, it has been suggested that Nrf2 also protects cells from radiotherapy, chemotherapeutic agents, and anticancer drugs through its antioxidant defense mechanism [20, 21]. Elevation of Nrf2 levels has been shown in clinical studies in cancer such as lung, ovarian, melanoma, colorectal cancer, endometrial carcinoma, breast cancer, kidney cancer, pancreatic cancer, endometrial carcinoma, and hepatocellular carcinoma [22]. Moreover, an increase in Nrf2 levels has been associated with therapeutic resistance and metastatic invasion in cancer cells [20]. The pharmacological role of Nrf2 has been demonstrated in studies with mice deficient in Nrf2 and single nucleotide polymorphism in the NRF2 gene NFE2L2 [23]. These and other findings have suggested that targeting the Nrf2 pathway may be a new cancer therapy. Researchers have tried to identify molecules activators of NrF2 as chemoprevention ROS-dependent carcinogenesis, while others have focused on identifying NrF2 inhibitors to increase sensitivity of cancer cells to chemotherapy.

Due to the dual effect of Nrf2 as defends normal cells under oxidative stress and their role in the redox adaptation of the cancer cells. Therefore it is logical that both activators and inhibitors of Nrf2 could act in anticancer therapy [24]. It is widely accepted that chemoprevention in cancer includes the activity of detoxifying and cytoprotective enzymes. Using compounds capable of activating the NRF2 could be a possible strategy in cancer prevention and therapy [6]. However, due to the modulatory effects of Nrf2 in the detoxification process, the potential use of activators in cancer prevention and therapy needs to be further elucidated. In addition, a high expression of Nrf2 has been associated with a low response from radiation and some anticancer drugs therapy such as carboplatin, cisplatin, 5-fluorouracil, and doxorubicin [25, 26]. Considering the oncogenic nature of Nrf2, using Nrf2 inhibitors is an exciting approach for treating cancers with elevated Nrf2 levels and could be a reasonable alternative to control tumor growth. In this regard, Nrf2 inhibitors decrease drug-detoxifying enzymes, inducing an increase in chemotherapeutic sensitization [17]. The transcription factor Nrf2, which has long been thought to be the master regulator of cytoprotective responses against electrophilic/xenobiotic and oxidative stress, has recently been discovered to promote cancer growth, progression, and therapy resistance [27]. More research on Nrf2 has observed its function in various types of tumours and possible therapeutic approaches to prevent or reverse its activation. Nrf2 pharmacological regulation tends to be context-dependent in cancer [28].

Secondary metabolites in plants with a typical aromatic ring bearing one or more hydroxyl groups are known as phenolic compounds [29]. Simple phenols, flavonoids, lignins and lignans, tannins, xanthones, and coumarins are examples of phenolic compounds isolated from plant sources. Some of these phenolic compounds have been shown to have potent anti-cancer properties as well as the ability to fight oxidative stress-related diseases. Phenolic and sulfur-containing compounds are two main groups of natural compounds that show promise as chemopreventive agents [30, 31]. Phenolic compounds have a variety of biological properties, including anticarcinogenic activity [32]. One of the most significant molecular mechanisms for polyphenols' cancer chemo-preventive effects is the induction of phase II detoxifying and antioxidant protection enzymes via the Nrf2/Keap1 signalling pathway [22]. Curcumin and resveratrol have been investigated extensively as natural compounds with anticancer properties in a variety of cancers. Via interactions with multiple cell signalling proteins, various cancers' proliferation, invasion, angiogenesis, and metastasis are inhibited [6]. This comprehensive review aims to offer updates on the role of phenolic compounds as anticancer agents due to their inhibitory action on Nrf2 inhibition.

## Review methodology

The relevant information about the role of phenolic compounds as potential anticancer agents with inhibitory effects on Nrf2 signaling pathway were collected from electronic scientific databases such as PubMed/MedLine, Web of Science, Scopus, Google Scholar, ScienceDirect and SciFinder. For searching we used the next MeSH terms: “Antineoplastic Agents/pharmacology”, “Antioxidant Response Elements /drug effect”, “Drug Resistance”, “Neoplasm/drug effect”, “Drug Resistance, Neoplasm/genetics”, “NF-E2-Related Factor 2/antagonists & inhibitors”, “NF-E2-Related Factor 2/genetics”, “NF-E2-Related Factor 2/metabolism”, “Neoplasms/drug therapy”, “Neoplasms/genetics”, “Neoplasms/metabolism”, “Neoplasms/pathology”, “Oxidative Stress/drug effects”, “Proto-Oncogenes”, “Signal Transduction”. The scientific names of the plants have been validate according to the World Flora Online, and the chemical structures according to the PubChem [33, 34].The most representative data and mechanisms of actions were summarized in tables and figures.

## Phytochemistry of phenolic compounds

Medicinal plants used in folk medicine are known to be small pharmaceutical factories, which are capable of producing compounds with interesting biological properties, such as antioxidant, anti-inflammatory, antimicrobial, antihypertensive, anticancer, etc. [35–38]. To check the activity of biological molecules, present in medicinal plants, in the first, a step they have to be extracted and then characterized [39]. Water [40–44], ethanol [45–48], methanol [49–54], methanol: dichloromethane [43], *n*-hexane [41], chloroform [41], ethyl acetate [41], butanol [41] are mainly used as solvents for the extraction of phenolics with anticancer activities. Phenolic acids can be found in a variety of plant components, including roots, leaves, fruits, and vegetables. Caffeic acid is the most common type of phenolic acid contained in fruits, while ferulic acid is found in an esterified form in the cell walls of the seed coat, bran, and fruits [55, 56]. Plant leaves and stems have higher levels of phenolic acids, but there are major differences between species [57]. Complex polyphenols, for example, can be found in cell vacuoles, tissues in the leaf, epidermis, flowers, and fruits. Tannins are abundant in bark, wood, and fruit pods, while flavonoids are abundant in flowers. There are different varieties of phenolic compounds (flavonoids, hydroxybenzoate, coumarins, hydroxycinnamates, xanthones, chalcones, stilbenes, lignins, and lignans) and their metabolic pathways have been reported in several review articles [58, 59]. Phenolic compounds are generally divided into two groups:

i) simple phenols (phenolic acids and coumarins);
  Phenolic acids include hydroxybenzoic acids (e.g. gallic acid, *p*-hydroxybenzoic acid, protocatechuic acid, vanillic acid and syringic acid) and hydroxycinnamic acids (e.g., ferulic acid, caffeic acid, *p*-coumaric acid, chlorogenic acid and sinapic acid) [31]. The basic phenolic compounds with 6- and 9-carbon skeletons, benzoic acids, and cinnamic acids are found in nature. A carboxylic group is added to the benzene ring, which is followed by one or more hydroxyl or methoxys groups. Gallic acid, for example, has three hydroxy (-OH) groups attached to the third (meta), fourth (para), and fifth (meta) carbons, while syringic acid has two methoxys (-OCH_3_) groups at the third and fifth (meta) carbons, and one -OH group at the fourth carbon (para). Compounds with a higher molecular weight are known as complex phenolics. These phenolic acids are mostly present in the vacuoles of cells. Complex phenolics found in fruits and vegetables are best represented by tannins and flavonoids.
ii) polyphenols (flavonoids and non-flavonoids like tannins, lignans, and stilbenes).
  The flavonoids are categorized mainly into flavones (e.g. apigenin, luteolin), flavonols (e.g. quercetin, rutin, kaempferol), flavanones (e.g. naringenin, hesperetin), flavanols (e.g. catechin, epicatechin, gallocatechin, epigallocatechin), anthocyanins (e.g. cyanidin, malvidin, petunidin), chalcones (e.g. arbutin, phloretin), isoflavonoids (e.g. genistein, daidzein) [31, 60].

**Fig. 1:**
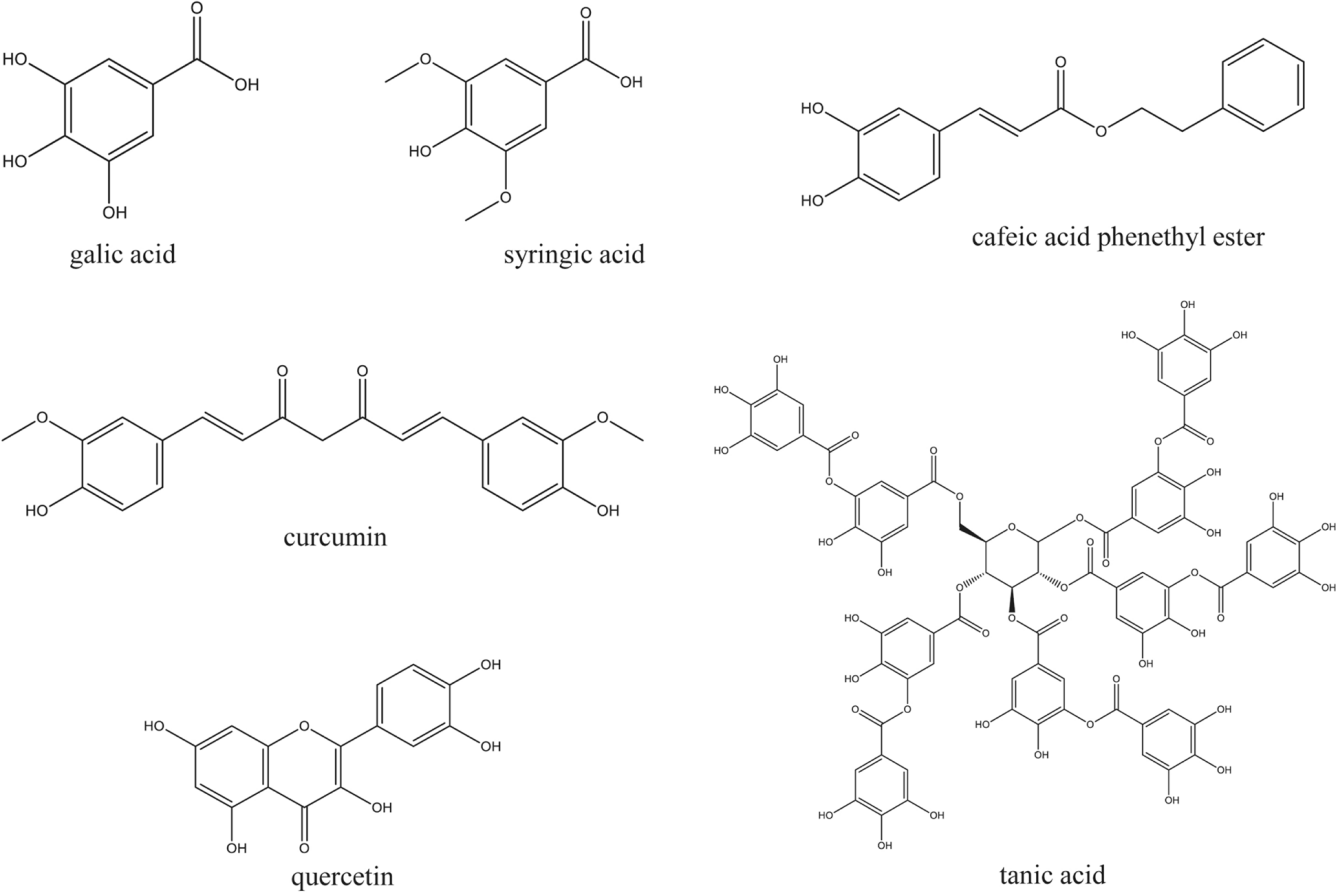
Chemical structures of bioactive phenolics compounds which are used as potential anticancer agents

Flavonoids are composed of two phenolic rings joined by an oxygenated heterocyclic pyran ring [61]. Flavonoids are further divided into anthocyanins, flavones, and flavanols based on the oxygenated state of the pyran ring. Beyond their main substitutions with hydroxyl or methoxy groups, these molecules are acetylated or glycosylated to achieve higher complexity [62]. Some of the naturally-occurring bioactive phenolics compounds which are used as anticancer agents are summarized in Fig. 1.

## Naturally-occurring polyphenolic compounds as anticancer agents

### Ethnomedicinal and traditional importance

Herbal medicine, also known as botanical medicine, phytomedicine or phytotherapy is one of the oldest and continues to be of great interest [63, 64]. Folk medicinal applications of traditional remedies are popular in many countries all over the world [65]. This natural medicine is usually used by people who want to improve their health and do not have access to conventional medical services [66, 67]. Most often plant-derived products such as vegetables, herbs, spices, and fruits are used as natural remedies or food additives [68] Usually, these plants are applied in the form of poultice, decoction, infusion or concoction, but the most commonly used form is water extracts [49, 69, 70]. Traditional medicine is a collection of traditional, natural recipes based on beliefs and experiences indigenous to different cultures [71, 72]. A wide variety of fruits, vegetables, herbs, species, flowers, trees, and bushes is known in folk medicine as a source of phytochemicals, which are used to treat health-related ailments and their associated symptoms [73, 74]. It is well known that cancer diseases are linked to an imbalanced diet poor in fruit and vegetables [75–77]. They have been used for centuries as natural remedies all over the world Recently, dietary phenolics have aroused great interest in the prevention and treatment of cancer [31, 60, 78–80]. Phenolic compounds are known to be responsible for their chemopreventive properties (e.g., antioxidant, antimutagenic and anti-inflammatory effects) [31, 51, 81, 82]. Aumeeruddy, Mahomoodally [83] in their documentation of medicinal plants, traditionally used in cancer management (performed for years1980-2019), showed that in a total of 62 countries, 948 plant species from 153 families and 628 genera were reported to be used against cancer. The most popular were vegetables – onion *(Allium cepa* L.), spices* –* ginger *(Zingiber officinale* Roscoe), turmeric *(Curcuma longa* L.) and black cumin *(Nigella sativa* L.), herbs – nettle (*Urtica dioica* L.) and black calla *(Arum palaestinum* Boiss.), fruits – pomegranate *(Punica granatum* L.) and graviola *(Annona muricata* L.) and shrubby succulent plant *(Aloe vera* (L.) Burm.f.). In the literature, there are numerous reports from in vitro, but also in vivo experiments confirming the effectiveness of traditional plant-based remedies rich in phytochemicals in the prevention or mitigation of human cancers (mainly due to anti-proliferative and apoptotic effects). Allium genus vegetables, such as garlic (*Allium sativum* L.), *A. cepa*, shallot (*Allium hirtifolium* Boiss.), leek (*Allium tuberosum* Rottler ex Spreng.) and chives (*Allium schoenoprasum* L.) have been exploited in folk medicine for centuries and are proposed to prevent cancers [84]. Vegetables from *Cruciferous* family like cauliflower, cabbage, Chinese cabbage, broccoli, Brussels sprouts, Pak choi are a popular component of the diet, consumed in large quantities. They possess strong antioxidant potential due to secondary metabolites like phenolics [85]. Moringa (*Moringa oleifera* Lam.) is another common vegetable, used in many countries since ancient times, due to a wide array of biological activities attributed to phenolic compounds [41]. According to a recent study, one of the most popular in cancer management is *U. dioica* and *A. palaestinum*. The cancer chemopreventive potential of nettle (especially in the case of breast cancer) was described in detail in the review of Esposito et al. [86] and Hodroj et al. [42]. The authors presented numerous examples of the use of *U. dioica* extracts (obtained with water, ethanol, methanol, and dichloromethane) in the in vitro treatment of breast, cervical, epidermoid, colon, gastric, lung, and prostate cancer cells. *A. palaestinum* is traditionally used in Palestinian herbal medicine for the treatment of diverse diseases like stomach acidity, atherosclerosis and also cancer. Its effectiveness is attributed, inter alia, to polyphenols – mainly flavonoids [43, 45].

Spices like *Z. officinale*, *C. longa*, *N. sativa* and many others contain polyphenols and can act as anticancer agents. Ginger is a common spice in food (used as a vegetable), beverages (e.g. tea) and herbal medicine, which is rich in bioactive phenolics, such as gingerols, paradols and shogaols. Is proposed to be used in the treatment of breast, colorectal, and prostate cancer cells [87]. Turmeric – an aromatic and nutraceutical plant, intensively used in Indian traditional medicine (Ayurveda), contains curcumin – a polyphenolic compound, which can be used in the treatment of nasopharyngeal, lung, hepatobiliary, breast, gastric, colorectal, uterus, prostate cancer and hematopoietic tumor [47, 87]. An effective therapeutic potential of black cumin to suppress tumor development, reduce tumor incidence and ameliorate carcinogenesis was described by Majdalawieh, Fayyad [88]. As shown in this review, black cumin seed extract induces cytotoxic effects against human hepatoma, adenocarcinoma, breast cancer, Dalton's Lymphoma Ascites, Ehrlich Ascites Carcinoma etc. Another spice that is widely used in many dishes is black pepper (*Piper nigrum* L.), which exhibits anticancer properties against several cell lines like breast, cervical, prostate and colon [89]. Polyphenols derived from cinnamon (*Cinnamomum cassia* (L.) J.Presl) – a traditional oriental medicinal herb, are examined for the treatment of melanoma, leukemia, etc. [87]. Besides vegetables, herbs and species, also fruits can be a rich source of antioxidants in the daily diet and can exert chemopreventive and/or chemotherapeutic potential [90]. According to Aumeeruddy, Mahomoodally [83], the most often examined fruits, in terms of anticancer properties are *P. granatum* and* A. muricata*. The anticancer activity of pomegranate is generally attributed to the high polyphenols content [91]. In the comprehensive review of Turrini et al. [91], prevention and treatment methods for several types of cancer cells (breast, prostate, lung, colon, leukemia, bladder cancers, and brain tumors) were described. The second popular fruit is graviola*,* a fruit tree with a long history of traditional use in folk medicine*,* especially in Africa and South America [92]. The authors in their review presented plenty of studies showing anti-proliferative effects of different extracts (water, ethanol, ethyl acetate) obtained from *A. muricata* towards various cancer cell lines, like as example lung A549 cancer cells, colon HT-29 cancer cells, K562 chronic myeloid leukemia cells, metastatic breast cancer. Fruits such as blueberries, strawberries, kiwi, and apples can also contain another polyphenolic compound with potent antioxidant properties – catechins (flavanols of the flavonoid family). But the most commonly consumed source of catechins in green tea – natural health beverage. Catechins extracted from green tea (*Camellia sinensis* (L.) Kuntze) are proposed to be efficient in the prevention of lung, breast, esophageal, stomach, liver and prostate cancer [80]. Biomedical potential in cancer prevention, control and reduction of development may also have seaweeds (also known as macroalgae), which are widely used in Asia, especially in traditional Chinese medicine and Japanese folk medicine, as well as a component of the daily diet [52, 53, 93]. Polyphenol-rich seaweeds in cancer research can exert antioxidant, antiangiogenesis effects, anti-proliferative efficacy (breast cancer cell lines), as well as can induce apoptosis [52, 53]. Shrubs and trees, traditionally used in folk medicine, can also constitute candidates for the isolation of novel bioactive compounds with anticancer properties [51, 94]. Phenolic compounds can be found in all parts of plants like roots, bark, lignified parts, leaves, flowers, fruits and seeds [94]. For example, *Ficus is* one of the largest genera of medicinal plants all over the world in tropical and subtropical regions – there are around 750 species of woody plants, trees and shrubs rich in polyphenols [51]. Trees and shrubs growing in a temperate climatic zone – pine, spruce, fir, beech, oak, walnut, willow, rowan, hawthorn, and bird cherry also offer a wide variety of phenolic compounds like phenols, phenolic acids, phenylpropanoids, flavonoids, coumarins, tannins [94]*.* Shrubby succulent plant – *A. vera* is one of the few botanical medications, which is in widespread domestic use. This plant is considered a good natural source of antioxidants. In folk medicine, aloe juice, as well as fresh leaf gel, are used in many nutraceutical and cosmetics formulations [95].

### From medicinal plants to potential anticancer bioactive compounds

The secondary metabolites, extracted from plants may be responsible for antioxidant and anticancer properties [96, 97]. Phenolic compounds are effective scavengers of ROS and RNS including the scavenging of superoxide anion, hydrogen peroxide, nitric oxide, free radical 2,2-diphenyl-1-picrylhydrazyl (DPPH) and 2,2′-azino-bis(3-ethylbenzothiazoline-6-sulfonate) radical cation (ABTS) [89]. Free radicals are known to be potential carcinogens [50], therefore, antioxidants used by cancer patients can help maintain the balance between the free radicals formed and the trapping abilities of radicals [50]. Natural compounds derived from dietary vegetables and fruits receive considerable attention for the prevention and treatment of cancer. *Allium* genus vegetables like *A. sativum*, *A. cepa*, *A. tuberosum*, *A. schoenoprasum* and *A. hirtifolium* are one of the most interesting antioxidants in the prevention of cancer. Asemani et al. [84] in their review summarized the anticancer activities of biologically active compounds derived from *Allium* genus vegetables. Phenolic acids like gallic and ferulic acid, as well as flavonoids (flavonols – quercetin, kaempferol, myricetin; flavones – apigenin, luteolin; flavanols – catechin, epicatechin) and tannins are predominant phenolics found in *Allium* vegetables. All of the listed vegetables are strong antioxidants, participate in ROS scavenging and exert cytotoxicity and apoptosis‐inducing effects – for example, garlic – induction of apoptosis in human leukemia cell line (HL‐60), human erythroleukemic cell line (OCIM‐1), chronic myeloid leukaemia cells (CML), liver cancer cell lines – human hepatoma (HepG2) and Hep3B cells. Onion showed cytotoxic and anti-proliferative effects on a breast cancer cell line (MCF‐7), human breast adenocarcinoma cell line (MDA‐MB231), HL‐60, HepG2, human colon cancer cell line (HT29) and prostate cancer (PC3) cells. Popular *M. oleifera*, used as a vegetable and a popular plant in traditional herbal medicine, contains several phytochemicals including phenolic compounds like flavonoids – flavonols (e.g. quercetin), phenolic acids (e.g. gallic acid, sinapic acid, vanillic acid, caffeic acid and syringic acid), which exhibit various biological activities such as antioxidant, antimicrobial, antiviral, anti-inflammatory, immune-boosting, antidiabetic, antiatherosclerotic and anticancer (e.g. cervical carcinoma) [41, 98]. Herbs are known to be a rich source of phenolic compounds in traditional medicine. For example, *U. dioica* owes its biological properties (e.g. antioxidant, antimutagenic and anti-proliferative) to phytonutrients such as phenolic compounds, including coumarins and polyphenols – flavonoids (flavonols – myricetin and rutin), tannins and lignans [86]. Hodroj et al. [42] also showed that the chemical composition of *U. dioica* aqueous extract indicated the presence of flavonoids, mainly patuletin, rutin, quercetin, apigenin and phenolic acids as caffeic acid, m/p-hydroxybenzoic acid, gallic acid, syringic acid to which the pro-apoptotic and antitumor properties can be attributed. Fern’s (*Asplenium nidus* L.) bioactive compounds, especially flavonoids, exhibit antioxidant, antibacterial (against multidrug-resistant pathogens like *Pseudomonas aeruginosa*, *Proteus vulgaris* and *Proteus mirabilis*) and anticancer properties (e.g. human hepatoma and human carcinoma) [54]. Spices commonly used in many cuisines all over the world can easily provide phenolic compounds in the diet. Black cumin seed extract, containing polyphenols like flavonoids (quercetin, an important flavonol) exerts numerous activities such as anti-proliferative and pro-apoptotic effects (reduction in serum levels of total sialic acid, lipid-bound sialic acid, α-fetoprotein, tumor necrosis factor (TNF-α), interleukin-6 (IL-6), prolactin, estradiol, progesterone, tissue caspase-3, -8, -9 activity), antioxidative and cytotoxic effects (reduced serum levels of malondialdehyde (MDA), nitric oxide, ROS, reduction in lipid peroxides, increased level of glutathione (GSH)), antimutagenic effect (increase in detoxifying enzymes, degradation of mutagens, DNA repair) [88]. Phenolic compounds (e.g. 3,4-dihydroxyphenyl ethanol glucoside, 3,4-dihydroxy-6-(N-ethylamino)benzamide and phenolic acid glycosides) extracted from medicinal plants like *P. nigrum* can scavenge DPPH radicals [99]. *C. cassia* extract due to the content of polyphenols (e.g. tannins), as well as essential oils (e.g. cinnamic and cinnamyl aldehyde) and carbohydrates, shows various biological functions including antioxidant, antimicrobial, anti-inflammatory, antidiabetic, anti-apoptotic and antitumor activity (e.g. cervical carcinoma and epithelial colorectal adenocarcinoma) [40, 87]. Besides vegetables, also fruits contain natural substances, such as antioxidants that have cancer-protective effects. *P. granatum* is one of the fruits, which has been used for the prevention and treatment of many diseases for centuries. Due to its strong antioxidant activity, it was proposed as a promising chemopreventive/chemotherapeutic agent. The anticancer activity of pomegranate is generally attributed to the high content of polyphenols such as ellagitannins, ellagic acid, and other flavonoids (flavonols – quercetin, kaempferol, flavones – luteolin) [100]. Phytochemical evaluation of *A. muricate* showed the presence of various phytochemicals such for example phenolics – hydroxycinnamic acids (e.g. *p*-coumaric acid, caffeic acid) and flavonol triglycosides, which can be responsible for antiparasitic, antimalarial, anticonvulsant, anti-arthritic, hepatoprotective, antidiabetic and anticancer activities [92]*.* A Brazilian berry – jaboticaba (*Plinia cauliflora* (Mart.) Kausel) is traditionally used in folk medicine. Phenolic compounds such as flavonoids, anthocyanins, ellagitannins are responsible for their antioxidant, anti-inflammatory, acetylcholinesterase, antifungal and chemo-preventive activities [48]. Drinking green tea is suggested to have many beneficial health effects, including cancer prevention. Catechins (flavan-3-ol) like epicatechin, epigallocatechin, epicatechin gallate and epigallocatechin gallate, belonging to the group of flavonoids from *C. sinesis* strongly neutralize ROS and RNS, inhibit the formation of free radicals and lipid peroxidation [80]. Seaweeds (brown, green and red macroalgae) are known as a rich source of bioactive phenolic compounds such as phlorotannins (found only in brown seaweeds), bromophenols, flavonoids, phenolic terpenoids and mycosporine-like amino acids, which exhibit a wide range of activities such as antioxidant, anti-inflammatory, anti-allergic, antibacterial, antiviral, antidiabetic, hepatoprotective, hypotension, neuroprotective and anticancer (e.g. breast cancer) [52, 53, 93]. Lopez et al. [95] examined the phenolic profile of extracts derived from the *A. vera* leaf skin and flowers. Catechin, sinapic acid, quercetin, quercitrin, rutin, myricetin and epicatechin were the main compounds found in leaves, whereas gentisic acid, epicatechin and quercitrin, sinapic acid, gallic acid and rutin were the most prominent phenolic compounds in flowers. Additionally, both extracts exhibited strong antioxidant activity. *Senegalia macrostachya* (Rchb. ex DC.) Kyal. & Boatwr., due to the presence of polyphenols like tannins, as well as triterpenes, steroids may be responsible for antioxidant and anticancer properties (e.g. acute and chronic myeloid leukemia) [50]. *Ficus carica* L. tree is known to contain phenolic acids, flavones and flavonols (quercetin) and inhibit the growth of human cervical carcinoma, liver and breast cancer cells [101]. Biologically active compounds derived from medicinal plants may be an alternative in the search for new anticancer molecules. The examples of medicinal plants used in folk medicine and their biologically active phenolic compounds with anticancer properties (cytotoxic effect) are presented in Table 1.

**Table 1:** Examples of medicinal plants as a source of phenolic compounds with anticancer properties

**Medicinal plants**	**Bioactive compound-phenolics/type of tested extract**	**Experimental Model**	**Results**	**Ref**
*Moringa oleifera* (moringa, *Moringaceae*)	Quercetin, phenolic acids: gallic, sinapic, vanillic, 4-hydroxy-3-methoxy benzoic, *p*-coumaric, *m*-coumaric, 4-hydroxy-3-methoxy cinnamic, caffeic, syringic *n*-hexane extract	in vitro Hela cervical cancer cells	↓ cancer cell viability IC_50_ = 416 μg/ml	[41]
*Capsicum annuum* (sweet pepper, *Solanaceae*)	Phenolics – flavonoids dihydroxycinnamic acids methanolic and ethanolic extract	in vitro PC3 prostate cancer cells L929 normal fibroblasts HCT116 colon carcinoma cells	↑cytotoxicity IC_50_ = 51 μg/ml no toxicity on L929 IC_50_ = 94 μg/ml ethanolic extract which contains capsianoside, and its fractions – water, 40% methanol in water and 70% methanol in water were less cytotoxic for HCT116	[46]
*Urtica dioica* leaves (nettle, *Urticaceae*)	Flavonoids and phenolics, mainly patuletin, m/p-hydroxybenzoic acid, caffeic acid aqueous extract	in vitro U937, KG-1 acute myeloid leukemia cells	↓proliferation IC_50_ = 40 μM–100 μM/ml	[42]
*Arum palaestinum* (*black calla,* Araceae)	Polyphenols—flavonoids ethanolic extract	in vitro Hela cancer cells	↑cytotoxicity, ↓proliferation IC_50_ = 256 -512 μg/ml	[43]
*Arum palaestinum* (*black calla,* Araceae), *Urtica pilulifera* (nettle, *Urticaceae*)	Phenols—flavonoids ethanolic extract	in vitro MCF-7 breast cancer cells	↑cytotoxicity antioxidant, free radical scavenging *U. pilulifera* extract, IC_50=_ 63 μg/ml *A. palaestinum* extract, IC_50=_ 500–600 μg/ml	[45]
*Asplenium nidus* (fern, *Aspleniaceae*)	Flavonoids (gliricidin7-*O*-hexoside, quercetin-7-*O*-rutinoside, keampferol-3-*O*-rutinoside, myricetin-3-*O*-rhamnoside) ethanolic extract	in vitro HepG2 human hepatoma cells Hela cervical carcinoma cells	↑cytotoxicity antioxidant free radical scavenging	[54]
*Curcuma longa* (turmeric) and *Zingiber officinale* (ginger) (*Zingiberaceae*)	Polyphenols ethanolic extract	in vitro B164A5 murine melanoma cells	↑antioxidant capacity and amount of polyphenols in ethanolic extract from *C*. *longa* rhizome than from *Z. officinale* rhizome; inhibition index for the B164A5 cell line two times was higher for *Curcuma* compared with *Zingiber* extract	[47]
*Cinnamomum cassia* (cinnamon, *Lauraceae*)	Polyphenols—tannins ethanolic extract	in vitro B16F10, clone M3 mouse melanoma cells Hela cervical carcinoma cells Caco2 human epithelial colorectal adenocarcinoma cells	↓proliferation IC_50_ = 0.5 mg/ml	[40]
*Senegalia macrostachya* (*Fabaceae*)	Flavonoids—tannins dichloromethane and methanolic extract from the root and steam bark	in vitro U937, K562 myeloid leukemia cancer cells	antioxidant the root bark dichloromethane and methanolic extracts demonstrated higher cytotoxicity on U937 and K562 the stem bark methanolic extract selectively affected U937	[50]
*Plinia cauliflora* (berry, *Myrtaceae*)	Flavonoids, anthocyanins, ellagitannins ethanolic extract	in vitro L929 murine non-cancer cells MDA-MB-231 estrogenic receptor-negative breast cells	antioxidant non-toxic effect on L929, ↑ cytotoxicity ↓viability of cells IC_50_ = 500–1000 μg/ml	[48]
Green tea	Catechins—epigallocatechin gallate, epigallocatechin, epicatechin, epicatechin gallate ethanolic extract	in vitro MCF-7 breast cancer cells HepG2 liver cancer Hela cervical cancer cells PC3 prostate cancer cells A549 lung cancer cells	↑ cytotoxicity IC_50_ = 292 mg/ml for MCF-7 IC_50_ = 327 mg/ml for HepG2 IC_50_ = 331 mg/ml for Hela IC_50_ = 351 mg/ml for PC3 IC_50_ = 384 mg/ml for A549	[79]
*Eucheuma cottonii* (red seaweed, Rhodophyta)	Phenolics—catechin, rutin, quercetin	in vitro MCF-7 estrogen-dependent human breast cancer cells MB-MDA-231 estrogen-independent human breast cancer cells	↓proliferation IC_50_ = 20 μg/ml for MCF-7 IC_50_ = 42 μg/ml for MB-MDA-231 no toxicity on normal cell lines	[52]
*Sargassum muticum* (brown seaweed, Ochrophyta)	Phenolics – catechin, phlorotannin, quercetin methanolic extract	in vitro MCF-7 estrogen-dependent human breast cancer cells MB-MDA-231 estrogen-independent human breast cancer cells	↑ cytotoxicity ↑apoptosis IC_50_ = 22 μg/ml for MCF-7 IC_50_ = 55 μg/ml for MDA-MB-231	[53]
*Acanthospermum hispidum* (bristly starbur, *Asteraceae*)	Polyphenolics methanolic extract	in vitro MCF-7 breast cancer cells CORL2 human large cell lung carcinoma cells SVK-14 normal human keratinocytes	↑ cytotoxicity IC_50_ ≤ 50 μg/ml	[49]
*Diospyros kaki* (persimmon)	Polyphenols Kaki tannin water extract	in vitro Molt 4B human lymphoid leukemia cells	↓ growth of cancer cells ↑ apoptosis	[44]
*Ficus carica* (fig tree, *Moraceae*)	Polyphenols – flavonoids Tannins methanolic extract	in vitro Huh7it liver cancer cells	antioxidant ↓ growth of cancer cells IC_50_ = 1000 μg/ml	[51]

## Phenolic compounds in cancer prevention and treatment: impact on Nrf2

**Fig. 2:**
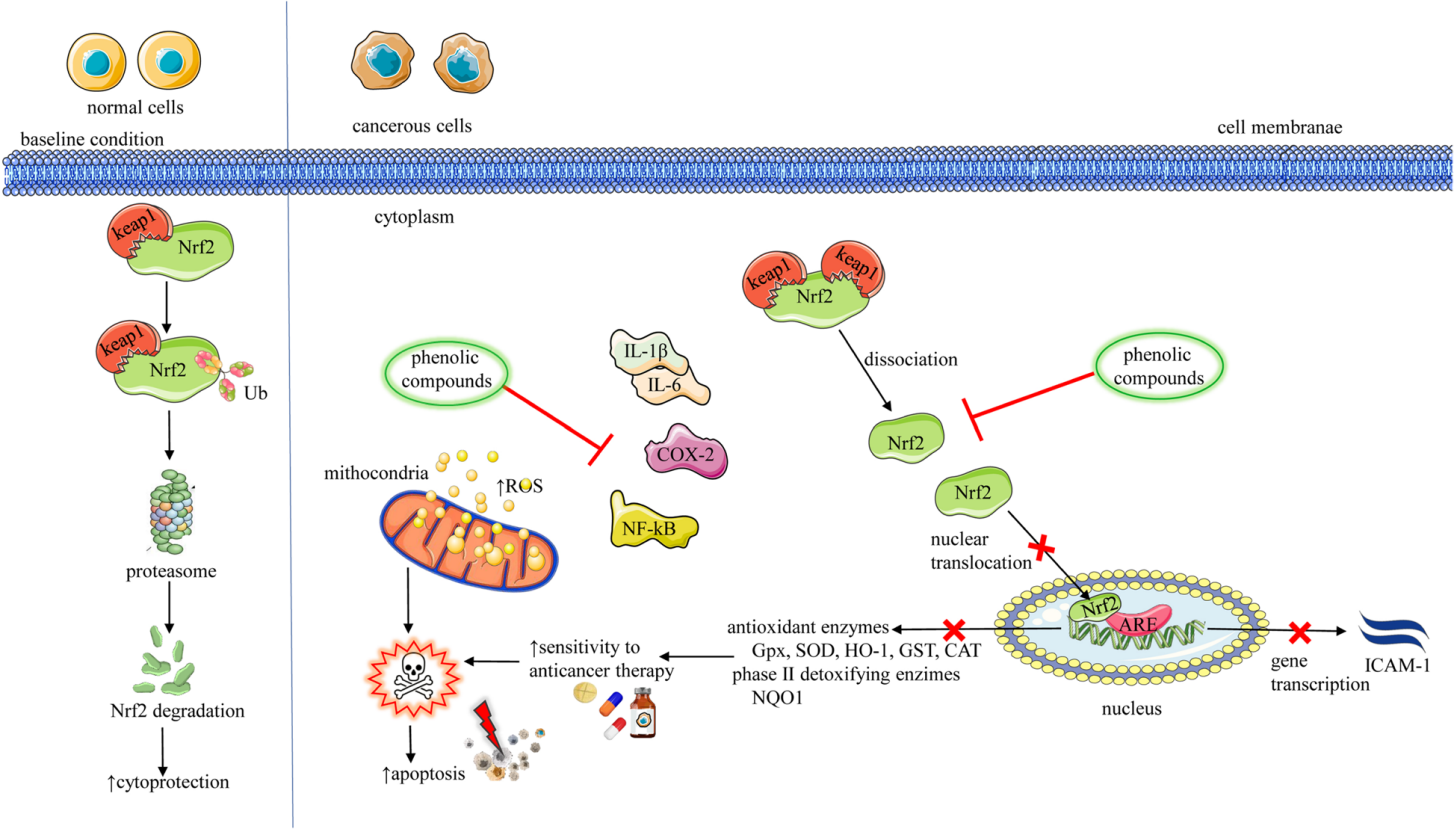
Summarized scheme regarding the inhibitory effect of phenolic compounds on Nrf2. Nrf2 inhibition by phenolic compounds leads to increasing the sensitivity of cancer cells to conventional anticancer therapy, reducing tumor growth and cancer cells death. [105] Abbreviations and symbols: ↑ increase, ↓ decrease, Nrf2 (nuclear transcription factor), ROS (Reactive oxygen species), Nrf2-Keap 1 (Kelch-like ECH-associated protein 1), Ub (Ubiquinone), NF-κB Nuclear factor kappa-light-chain-enhancer of activated B cells, Cox-2 (Cyclooxygenase-2), NQO1 (NAD(P)H quinone oxidoreductase 1), (GPx) Glutathione peroxidase, JNK Jun N-terminal kinases, ICAM-1 (Intercellular Adhesion Molecule 1)

The potential of phenolic compounds within natural extracts and on their own as new chemopreventive agents and/or cancer treatments is discussed below. The most important mechanisms of action are summarized in Table 2 and Fig. 2.

**Table 2:** Summarized data of the most representative results from preclinical studies regarding inhibitory effects of phenolic compounds on Nrf2 signaling pathways

Tested extracts/ polyphenolic compounds	Experimental Model	Main results	Ref
*Cinnamomi Cortex* extract	A549 human lung cancer cells	↓Nrf2 overexpression ↓IGF-1R ↓anticancer drug resistance	[102, 103]
*Chrysanthemum naktongense* Nakai licorice *Glycyrrhiza uralensis* Fisch	HT-29 human colon cancer cells HepG C8 human hepatoma cells	antioxidant ↑detoxifying enzymes induction mediated by ↓Nrf2 ↓NF-κB, ↓pro-inflammatory markers ↑NQO1, ↑Nrf2, ↑UGT1A1, ↑Nrf2-ARE	[104]
*Castanea crenata* Siebold & Zucc extract	MCF-7 breast cancer stem cells	↓Nrf2 ↑susceptibility of cancerc cells to anticancer drugs	[105]
rosemary extract	HT-29 human colon cancer cells	↓Nrf2, ↑ ROS ↑apoptosis, ↑cytotoxicity	[106]
strawberry tree honey	HT-29 human colon cancer cells	↓NF-kB, ↓P-IkBa, ↓Nrf2	[107]
*B Bergenia pacumbis* (Buch.-Ham. ex D.Don) C.Y.Wu & J.T.Pan extract	PC3 prostate cancer cells PC3 tumor xenograft mice	↓Nrf2 ↑ apoptosis, ↓MAO-A, ↑ROS ↓tumor growth	[108]
*Rhododendron luteum* Sweet extract	HeLa human cervical cancer cells	↓Nrf2, ↓ mRNA ↑antiproliferative effect	[109]
Luteolin	NHBE normal human bronchial epithelial NHBE cells exposed to cigarette smoke	IC_50_ = 5–40 μM ↓toxic effects of cigarette smoke ↑cell viability, ↓oxidative stress, ↓apoptosis ↓Nrf2, ↓NADPH, ↓NQO1, ↓HO-1	[110]
Wedelolactone isolated from *Eclipta prostrate* Lour		IC_50_ = 2.5–20 μM ↑ NHBE cell viability ↓COX-2, ↓ICAM-1 ↓NQO1, ↓HO-1	[111]
Procyanidins Isolated from *Cinnamomi* cortex	PC3 prostate cancer cells	IC_50_ = 2.5 μg/ml ↓Nrf2 ↑antiproliferative effect	[102, 112]
neferine isolated from *Nelumbo nucifera* Gaertn. leaves	KYSE30, KYSE150 and KYSE510 esophageal squamous cells carcinoma	IC_50_ = 0–20 μM ↓Nrf2, ↑ ROS, ↑JNK ↑antiproliferative effect	[113]
schisantherin A isolated from *Fructus schisandrae*	MKN45, SGC-7901 gastric cancer cell lines	IC_50_ = 2.5 μM ↓cell viability, ↓Nrf2 ↑cell cycle arrest, ↑apoptosis ↓migration, ↑ ROS/JNK	[114]
galloyl glucoses—1,2,3,4,6-penta-*O*-galloyl-β-D-glucose 1,3,6 -tri-*O*-galloyl-β-d-glucose isolated from *Excoecaria formosana*	Huh7 cancer liver cells	↓Nrf2 ↑antiproliferative effect	[115]
agrimoniin isolated from *Agrimonia pilosa* Aitch	PANC-1, CFPAC-1 pancreatic cancer cells	IC_50_ = 100, 200, 300 μM ↓Nrf2, ↑ ROS ↑apoptosis	[116]
pterostilbene a natural dimethoxylated analog of resveratrol	Tumour xenograft mice with human melanomas	↓Nrf2 ↓tumor growth of melanomas	[117]

*Abbreviations* and symbols: ↑increase, ↓decrease, *Nrf2 *Nuclear factor erythroid 2-related factor, *IGF-1R *Insulin-like growth factor 1 receptor, *NF-κB *Nuclear factor-kappa B, *NQO1* (NAD(P)H quinone oxidoreductase 1, *NADPH *Nicotinamide adenine dinucleotide phosphate, *HO-1* Heme oxygenase 1, *ROS *Reactive oxygen species, *JNK *Jun N-terminal kinase, *ICAM-1* Intercellular adhesion molecule

### Phenolic compounds-rich matrices with inhibitory effects on Nrf2

*Cinnamomi cortex* extract (CCE) has been shown to inhibit the nuclear translocation of Nrf2, thereby reducing the resistance of tumour cells to conventional chemotherapeutic drugs. Also, the results of this in vitro study performed on human A549 lung cancer cells showed that by inhibiting Nrf2 at the molecular level, CCE potently inhibits the enzyme NQO1 [102] (Fig. 2). In 2017, a novel mechanism by which CCE procyanidin was investigated in A549 cells, this study found that treatment can promote the degradation of nuclear Nrf2 through insulin-line grow factor-1 receptor (IGF-1R). Research results showed that the natural flavonoids contained in CCE suppress Nrf2 activity by inhibiting its nuclear translocation and Nrf2 mRNA expression, thus leading to increased sensitivity of cancer cells to the action of conventional cytostatic drugs [103] (Fig. 2). *Chrysanthemum naktongense* Nakai and licorice *Glycyrrhiza uralensis* Fisch. extracts presented an induction to antioxidant and detoxifying enzymes induction mediated by Nrf2. Both extracts inhibited NF-kB and proinflammatory markers such as COX-2, interleukins IL-6, IL-1β having antioxidant and anti-inflammatory effects; they also induced Nrf2 mRNA transcription, ARE expression, NQO1, UGT1A1 genes and phase II detoxifying enzymes leading to apoptotic death of cancer cells. Therefore, the extracts contribute to promoting pharmacological effects against diseases, like cancer [104].

Moreover, it has been observed that the extracts of *Castanea crenata* Siebold & Zucc. (chestnut) could rise, through the inhibition of the Nrf2 signaling pathway, the susceptibility of breast cancer stem cells (CSCs) to an anticancer drugs, which could be used as an adjuvant for chemotherapy. The mechanisms of action were to increase intracellular ROS levels, inhibit Nrf2-mediated intracellular antioxidant systems, thereby inducing apoptosis of cancer cells [105].

The antitumor activity of a rosemary extract (RE) in colon cancer cells has been investigated, which was obtained through supercritical fluid extraction. Treatment with RE caused an increment of intracellular ROS, leading to apoptosis of tumor cells. So, the cytotoxic effects of RE were increased by NRF2 gene silencing [106]. These results show that the ROS intracellular increment can target colon cancer cells and decrease cell survival mechanisms, which could be a potential treatment in colon cancer by the combination of chemotherapeutic drugs and rosemary compounds. It has been observed that strawberry tree honey (STH) can suppress the expression of pro-inflammatory markers such as NF-kB and P-IkBα **(**nuclear factor of kappa light polypeptide gene enhancer in B-cells inhibitor, alpha), and inhibition of Nrf2 in colon cancer cells. Glycolysis and mitochondrial respiration processes were also affected by STH, leading to apoptotic death of tumor cancer cells. So, STH could be helpful as a potential treatment for cancer prevention [107]. Another study evaluated the sensitizing effects of the polyphenol-rich fraction of *B Bergenia pacumbis* (Buch.-Ham. ex D.Don) C.Y.Wu & J.T.Pan in prostate cancer cells [108]. The treatment with the extract-induced apoptosis in PC3 cells both in vitro and in vivo on PC3-tumor xenograft mice involves in a process mediated by activation of monoamine oxidase A (MAO-A) catalytic activity, inactivation of Nrf2 pathway and increased ROS production. The combined treatment of the extract with Paclitaxel led to synergistic effects in reducing tumor growth and apoptosis of tumor cells. The effects of *Rhododendron luteum* Sweet extract (RLE) have been evaluated on human cervical cancer (HeLa) cells, and the results indicate that can inhibit the mRNA expression levels of Nrf2 showing an antiproliferative effect of RLE [109].

### Phenolic compounds with Nrf2 inhibitory effects

The effects of luteolin have been investigated in normal human bronchial epithelial (NHBE) cells exposed to cigarette smoke extract as the main causative agent of lung cancer [110]. Treatment with luteolin (5–40 μM) significantly reduced the toxic effects of cigarette smoke, improving cell viability and, reducing oxidative stress and inducing apoptosis. In addition, luteolin attenuated the protein expressions of Nrf2 and some of the downstream target proteins such as phase II enzymes NAD(P)H dehydrogenase: Quinone 1(NQO1) and heme oxygenase-1 (HO-1) (Fig. 2). Also, the effects of luteolin on Nrf2 were blocked when using a NRF2 gene knockdown model, evidencing the implication of the Nrf2 pathway in cigarette smoke toxicity. In a recent study using cigarette smoke, the treatment with wedelolactone (2.5–20 μM), isolated from *Eclipta prostrate* Lour., improved NHBE cell viability, recovered the antioxidant defence systems and reduced the levels of proinflammatory mediators such as cyclooxygenase 2 (COX-2) and intercellular adhesion molecule 1 (ICAM-1) [111] (Fig. 2). Moreover, wedelolactone also reduced the expression of NQO1 and HO-1, and this expression was blocked by treatment with the Nrf2 inhibitor all-trans-retinoic acid (ATRA). Procyanidins from *Cinnamomi* cortex (2.5 μg/ml) were found to exert anti-proliferative activity in lung and prostate cancer cells that overexpress Nrf2 and suppress the elevated Nrf2 expression and the Nrf2-regulated enzyme activity [102, 112] (Fig. 2). Further transfection assays demonstrated that procyanidins had a selective capability to inhibit the excessive activation of Nrf2. Another study reported that neferine (10–20 μM) from *Nelumbo nucifera* Gaertn. leaves induced apoptosis in esophageal squamous cell carcinoma by increasing ROS production and activating the c-Jun N-terminal kinase (JNK) pathway [113]. In addition, neferine inhibited the expression of Nrf2 favouring the production of ROS. The involvement of the Nrf2 pathway was confirmed by the pre-treatment of cells with the Nrf2 activator, tert‑butylhydroquinone (tBHQ), observing a partial compensation of the proliferative inhibition effect of neferine. The anti-proliferative effects of schisantherin A, a lignan isolated from *Fructus schisandrae* were investigated in gastric cancer cell lines MKN45 and SGC-7901 [114]. The treatment of cells with schisantherin A (2.5 μM) induced cell cycle arrest and apoptosis and inhibited cell migration. The pro-apoptotic effects were associated with the activations of ROS/JNK and the inhibition of Nrf2 pathways. In addition, treatment of cells with tBHQ counteracted the inhibitory effect of schisantherin A on cell viability activating the Nrf2 pathway. In a study where a total of 44 compounds were isolated from *Excoecaria formosana* Hayata & Kawak. ex Hayata was analysed, it was observed that two galloyl glucoses—1,2,3,4,6-penta-*O*-galloyl-β-D-glucose and 1,3,6 -tri-*O*-galloyl-β-d-glucose—significantly inhibited Nrf2 activity in cancer liver Huh7 cells [115]. Agrimoniin, a dimeric hydrolysable tannin isolated from *Agrimonia pilosa* Aitch., was also found to sensitize pancreatic PANC-1 and CFPAC-1 cells to apoptosis [116]. Agrimoniin treatment (100, 200, and 300 μM) increased the ROS production in pancreatic cells by suppressing the Nrf2 antioxidant signalling pathway, and altered energy metabolism leading to apoptosis. Regarding studies in animal models, the available data are still scarce and only one report was found. In that study, pterostilbene, a natural dimethoxylated analog of resveratrol, was investigated in tumour xenograft mice using different human melanomas [117]. Intravenous administration of pterostilbene decreased the growth of melanomas and downregulated the Nrf2-dependent signalling pathway after 35 days. In addition, a genetically induced Nrf2 overexpression in the melanoma cells blocked the inhibition of tumour growth.

## Synergistic effects of phenolic compounds associated with other anticancer agents on Nrf2

Promising studies have shown the ability of some phenolic compounds to sensitize cancer cells when combined with anticancer agents (Table 3). In this sense, chrysin (5,7-dihydroxy flavone) was investigated against doxorubicin-resistant BEL-7402 hepatocellular carcinoma cells [118]. The treatment with chrysin (10, 20 μM) significantly reduced the mRNA and protein levels of Nrf2 expression via down-regulation of the PI3K-Akt and ERK signalling pathways. The combined treatment with adriamycin and chrysin (10 μM) significantly enhanced the cytotoxicity when compared with adriamycin alone. Luteolin (3,4,5,7-tetrahydroxy flavone) was found to sensitize two resistant colorectal cancer cell lines (HCT116-OX and SW620-OX) to oxaliplatin [119]. Pre-treatment with luteolin (1, 5 and 10 μM) inhibited the Nrf2 pathway in a dose-dependent manner and also inhibited the expression of downstream target genes (HO-1, NQO1, GSTα1/2). In addition, luteolin when combined with chemotherapeutic drugs (doxorubicin, and cisplatin) resulted in a greater anticancer effect, indicating a synergistic effect. An interesting study revealed the capability of 3',4',5',5,7-pentamethoxyflavone, a natural flavonoid extracted from Rutaceae plants, to sensitize cisplatin-resistant A549 cells to cisplatin [120]. The treatment with the flavone (10–400 μM) ameliorated the elevated expression of Nrf2 and its downstream genes HO-1, NQO1 and cysteine ligase catalytic subunit (GCLC) in cisplatin-resistant A549 cells. Moreover, co-exposure of 3',4',5',5,7-pentamethoxyflavone with cisplatin-induced apoptosis to a greater extent than cisplatin alone and this effect was downregulated by siRNA specific for Nrf2. Another study reported the sensitizing capability of resveratrol (15 μM) when combined with clofarabine in mesothelioma MSTO-211H cells [121]. The combined treatment induced a noticeable growth-inhibitory effect, associated with the suppression of the Nrf2 pathway and decreased expression of HO-1. The role of Nrf2 was confirmed through overexpression of Nrf2 which conferred protection to cells and reduced the apoptosis rate. An interesting line of research is the one that deals with the sensitizing capacity of polyphenols in radiotherapy. There is evidence showing the radiosensitizing capacity of some polyphenols such as genistein, ferulic acid or alpinumisoflavone, among others (Wang et al. [122]). The mechanism of action in some of them is related to an increase in ROS production due to the inhibition of Nrf2-mediated cytoprotective gene expression.

**Table 3:** The Synergistic effects on Nrf2 of phenolic compounds associated with other anticancer agents

Type of anticancer agent/treatment associated with phenolic compounds	Experimental Model	Potential Mechanisms	Synergistic effect	Ref
Chrysin (5,7-dihydroxy flavone)	in vitro BEL-7402 doxorubicin resistant hepatocellular carcinoma cells IC_50_ = 10, 20 μM	↓ Nrf2 ↓mRNA ↓PI3K-Akt ↓ERK	sensitize cancer cells to doxorubicin ↑cytotoxicity	[118]
Luteolin (3,4,5,7-tetrahydroxy flavone)	in vitro HCT116-OX SW620-OX oxaliplatin-resistant colorectal cancer cells IC_50_ = 1, 5—10 μM	↓ Nrf2 ↓HO-1 ↓NQO1 ↓GSTα1/2	sensitize cancer cells to oxaliplatin ↑cytotoxicity	[119]
3',4',5',5,7-pentamethoxyflavone	in vitro A549 cisplatin-resistant lung cancer cells IC_50_ = 10–400 μM	↓ Nrf2 ↓siRNA ↓HO-1 ↓NQO1 ↓GCLC	sensitize cancer cells to cisplatin ↑apoptosis	[120]
resveratrol and clofarabine	in vitro MSTO-211H mesothelioma cells IC_50_ = 15 μM	↓ Nrf2 ↓HO-1	↓cancer cells growth ↑apoptosis	[121]
radiotherapy	-	↓ Nrf2 ↑ROS	radiosensitivity of cancer cells	Wang et al. [122]

## Therapeutic perspectives, challenges and limitations

Plant-derived phenolic compounds have great anticancer potential. They are responsible for chemopreventive properties such as antioxidant, antimutagenic and anti-inflammatory and the action of phenolic compounds can be considered on several levels, through different mechanisms [123, 124]. Phenolic compounds act as scavengers of ROS/RNS, which are potential carcinogens and induce the cellular defence antioxidant/detoxifying enzymes [125, 126] These compounds can induce antioxidant enzymes such as superoxide dismutase, glutathione-S-transferase, glutathione peroxidase and reductase (e.g., nordihydroguaiaretic in *Larrea tridentata* (DC.) Coville – [127]; green tea polyphenols – [80]). Polyphenols activate the Nrf2, which is responsible for the control of gene expression and regulation of antioxidant and detoxifying enzymes [80, 127]. As shown in Table 1, numerous plant-derived extracts exert a cytotoxic effect on different types of cancer cell lines. The lack of selectivity in the cytotoxic activity between cancerous and non-cancerous cell lines minimizes the perspective of using active compounds of plant origin as novel anti-cancer drugs [49].

Apoptosis induction is one of the strategies in cancer therapies. In the literature, it was shown that phenolic compounds can induce apoptosis by arresting the cell cycle, regulating carcinogen metabolism and ontogenesis expression, inhibiting DNA binding and reducing cell adhesion, migration, proliferation or differentiation, and blocking signaling pathways [128]. Such properties were noted for example for black pepper phenolics [89], catechin, phlorotannin, quercetin extracted from brown seaweed *Sargassum muticum* [53], nordihydroguaiaretic acid – a phenolic lignan from the leaves of the evergreen desert shrub (*L. tridentate*) [127], green tea polyphenols [79], persimmon (*Diospyros kaki* L.f.) extract in apoptosis of leukemia cells [44], cinnamon extract, which strongly inhibited tumor cell proliferation, induced active tumor cells death by up-regulating pro-apoptotic molecules and inhibiting nuclear factor κB (NF-κB) and activator protein 1 (AP-1) and their target genes such as BCL-2 and BCL-X_L_ – key regulators of apoptosis [40]. Cancer cell lines treated with phenolics-rich plant extracts can exhibit apoptotic morphological changes such as cell shrinkage, DNA fragmentations, a bulge of the plasma membrane of a cell, reduction or absence of microvilli, chromatin condensation and caspase cascade activation—major effector of apoptosis [52]. The main goal of the development of new therapeutic anticancer techniques is to elaborate a strategy to selectively induce apoptosis of cancer cells without altering healthy cells [44]. Based on potential applications in cancer treatment, several polyphenols have been characterized as inhibitors of Nrf2 circuits. For example, luteolin, and a 4-methoxy-chalcone derivative, have been promising in improving the sensitivity of tumor cells to neoplastic drugs [129, 130]. On the other hand, studies in cancer cells line showed that Camptothecin, another Nrf2 inhibitor, could be used in combination with other anticancer drugs to increase its efficacy in cancers with high Nrf2 levels [131].

Although the in vitro results of the use of Nrf2 inhibitors are promising, especially concerning sensitivity to chemotherapy of Nrf2-addicted cancers, the using systemic Nrf2 inhibitors may have pharmacological activity is unsatisfactory due to side effects caused by the essential roles of Nrf2 in cytoprotection. This aspect must be resolved before the use of Nrf2 inhibitors as drugs applied in cancer therapy. Moreover, challenges regarding efficacy, safety, pharmacodynamic properties, and target specificity remain. In this regard, still is under investigation therapeutic new molecules beside Nrf2 for tumors with high Nrf2 levels. In conclusion, the drug development that targets Nrf2 is moving slowly due to the dual effects of NRF2 in cancer.

In the case of medicinal plants, there is still a long way from folk medicine to clinical use. The observation of the natural environment and selection of the active substances that can alleviate disease symptoms should be verified and confirmed in the toxicological studies, which are associated with a detailed phytochemical analysis. It is important to evaluate the potentially toxic effects that may reduce the therapeutic value of natural remedies [132]. It is worth emphasizing that medicinal plants with anticancer molecules may support preventive and chemotherapeutic effects, but they cannot replace pharmacological treatment [80]. Scientific research conducted around the world on the cytotoxicity of extracts from medical plants on cancer cell lines confirms their effectiveness and supports their use in traditional medicine in cancer treatment. Also, experimental studies showed that the activity of plant extracts is usually attributed to the mixture of biologically active compounds (phenolic compounds, polysaccharides, proteins, amino acids, vitamins, minerals, phytosterols, essential oils, etc.), no single compounds. Therefore, the combined anticancer mechanism and effects of the various compounds, including phenolics on cancer cell lines have still to be explored.

## Conclusions

Natural products obtained from medicinal plants can serve as a rich source of compounds, for example, phenolic compounds with health-promoting properties [46, 50]. Due to antioxidant, anti-inflammatory and immunomodulatory properties, these compounds can be used in the prevention and treatment of several diseases, including cancer [87, 88]. Literature data have already confirmed the effectiveness of plant-derived products, including their cytotoxic effect on the cancer cell lines, which to a certain extent supports their traditional inclusion in cancer prevention and treatment [49]. The effect of natural products on the health maintenance and prevention of diseases is usually not confirmed by their exact chemical composition or the explained mechanism of action. Therefore, there is a need to find a relationship between the effectiveness of natural products and their composition and biological activity, confirmed in scientific research (in vitro and in vivo studies). Despite the enormous advances in conventional medicine and drug discovery, the use of herbal remedies is still extremely widespread around the world [88]. Nrf2 has a significant part in cellular defense against oxidative stress and exogenous toxic materials. Moreover, it plays a crucial role in tumorigenesis and drug sensitivity. Due to the critical role of Nrf2, it has emerged as a therapeutic target for the prevention and therapy of cancer. However, since Nrf2 has paradoxical roles in cancer biology, it is necessary to understand the molecular pathway leading to tumor suppress or the oncogenic effect of Nrf2 for the development of drugs with high specific and limited side effects. Natural products, including phenolic compounds, mediate the Nrf2/ARE pathway and may act as chemopreventive or chemotherapeutic agents. New phytodrugs need to be studied and developed to better understand the role of phenolic compounds in cancer.

## Data Availability

Not Applicable.
